# Corneal thickness measurements with the RTVue, Casia-2, and Pentacam devices in patients with mild-to-moderate keratoconus: a comparative study

**DOI:** 10.1186/s12886-023-02767-x

**Published:** 2023-01-26

**Authors:** Bingqing Sun, Xiaoyu Zhang, Ling Sun, Yangyi Huang, Mi Tian, Yang Shen, Lan Ding, Xingtao Zhou

**Affiliations:** 1 Eye Institute and Department of Ophthalmology, Eye & ENT Hospital, Fudan University, Shanghai, 200031, China; 2 NHC Key Laboratory of Myopia (Fudan University); Key Laboratory of Myopia, Chinese Academy of Medical Sciences, Shanghai, 200031, China; 3 Shanghai Research Center of Ophthalmology and Optometry, #83 FenYang Road/#19 Baoqing Road, Shanghai, 200031 People’s Republic of China; 4 Shanghai Engineering Research Center of Laser and Autostereoscopic 3D for Vision Care (20DZ2255000), Shanghai, China

**Keywords:** Corneal thickness, Keratoconus, Optical coherence tomography, Agreement

## Abstract

**Background:**

To compare the characteristics of corneal thickness measurements among the RTVue, Casia-2, and Pentacam in patients with mild-to-moderate keratoconus.

**Methods:**

We recruited 46 eyes of 46 patients diagnosed with mild-to-moderate keratoconus at our hospital between January and March 2022. The central corneal thickness (CCT) and thinnest corneal thickness (TCT) were measured using two optical coherence tomography (OCT) instruments (RTVue and Casia-2) and the more conventional Pentacam. Differences and correlations between the CCTs and TCTs, based on the device and influencing factors, were explored.

**Results:**

The CCTs were highly consistent among the groups (*p* = 0.434) and correlated with one another (*p* < 0.001). The TCTs measured by OCTs were thinner than those measured by the Pentacam (*p* < 0.001); however, all three devices were highly correlated (*p* < 0.001). The thinnest point location measurements with RTVue and Casia-2 differed significantly from the measurements with the Pentacam. Bland–Altman plots demonstrated a significant agreement between Pentacam and OCTs in TCT measurement (*p* < 0.001); the 95% limits of agreement were − 3.1 μm to + 33.1 μm for Pentacam and RTVue and − 8.6 μm to + 36.5 μm for Pentacam and Casia-2. RTVue and Casia-2 showed no difference in corneal thickness (*p* = 0.633) and thinnest point location measurement (*p* > 0.05). Multivariate analysis identified that the TCT measurement difference between the RTVue and Pentacam was related to the difference between the CCT and TCT (b = 0.490, 95% confidence interval [CI]: 0.033 to 0.948, *p* = 0.036), whereas the difference between the Casia-2 and Pentacam was related to the anterior radius for curvature (A) grade (b = 3.9, 95% CI: 1.753 to 6.074, *p* = 0.001), corneal pachymetry at the thinnest (C) grade (b = − 7.875, 95% CI: − 11.404 to − 4.346, *p* < 0.001), and the difference between the CCT and TCT (b = 0.425, 95% CI: 0.1 to 0.751, *p* = 0.012).

**Conclusions:**

CCTs in patients with mild-to-moderate keratoconus were similar among all three devices, but the TCTs and the thinnest point locations were not. Furthermore, the TCT measurement differences between the OCT devices and the Pentacam were more pronounced in keratoconus cases with a steeper anterior surface, thicker TCTs, and a larger difference between the CCT and TCT.

**Trial registration:**

Number: 2021118–1. Retrospectively registered: September 01, 2021.

**Supplementary Information:**

The online version contains supplementary material available at 10.1186/s12886-023-02767-x.

## Introduction

Keratoconus is a non-inflammatory, corneal ectasia disease characterized by chronic thinning of the central or paracentral corneal stroma and corneal apex protrusions, which lead to irregular astigmatism and high myopia. Furthermore, acute corneal edema occurs in the later stages, resulting in a corneal scar and permanent vision loss [1]. Therefore, measuring the corneal thickness is crucial for the early diagnosis, detection of disease progression, and treatment of keratoconus. Differences in the thinnest point locations and the corneal apex, as well as differences in the corneal thickness between the supranasal and inferior temporal areas, indicate early keratoconus [2]. According to the recent Belin and Duncan’s ABCD KC grading [3], keratoconus classification and progression are partly based on the corneal thickness. Furthermore, reliable measurement of the corneal thickness is necessary for monitoring corneal edema and endothelial function [4].

Corneal tomography, based on the Scheimpflug system (e.g., Pentacam), is currently the most commonly used clinical method to detect corneal thickness. It allows real-time examination of the anterior and posterior corneal surfaces, and its repeatability and reproducibility are widely recognized [5, 6]. Fourier-domain optical coherence tomography (FD-OCT) devices (such as the RTVue-100 and Casia-2) have provided a new method for evaluating the corneal morphology. These devices have increased penetration properties owing to their longer-wavelength light sources, which enable higher quality images, even in cloudy corneas [7]. These instruments also exhibit superior repeatability and reproducibility over the conventional Scheimpflug system since they acquire data faster [4, 8, 9]. In addition, FD-OCT devices can measure the corneal epithelial thickness and reveal early signs of keratoconus, such as corneal epithelial remodeling [10]. The FD-OCT instruments and the Scheimpflug system, which are commonly used in clinical practice, have different imaging mechanisms; thus, their results differ slightly. Many studies have shown that compared to the Pentacam, FD-OCT instruments tend to underestimate the corneal thickness in healthy eyes [11, 12]. However, few studies have compared the corneal thickness measurements between FD-OCT devices and the more conventional Pentacam or even among different FD-OCT devices in patients with keratoconus. Furthermore, none have investigated the factors affecting such differences.

Therefore, in this study, we used the RTVue and Casia-2 FD-OCT devices and the Pentacam to measure the central corneal thickness (CCT) and the thinnest corneal thickness (TCT) in patients with mild-to-moderate keratoconus to elucidate the characteristics of these three instruments.

## Methods

### Ethics

This study was conducted from January 2022 to March 2022 at the Eye and ENT Hospital of Fudan University, Shanghai, China, and followed the principles of the Declaration of Helsinki. This study was approved by the hospital’s ethics committee, and all participants signed an informed consent form (approval umber: 2021118–1).

### Patients

We recruited the following patients: 1) those with mild-to-moderate keratoconus diagnosed by an experienced clinician based on clinical manifestations and a tomographic examination [1] and 2) those whose Tomographic Keratoconus Classification stages (obtained from the Pentacam exam) were KC1–3 [13]. Patients who recently wore contact lenses (hard contacts within 4 weeks or soft contacts within 2 weeks) or those with an intraocular pressure > 21 mmHg, a family history of glaucoma, dry eye symptoms, corneal scarring, eye surgery history, or active ocular lesions were excluded [6, 14]. The sample size calculation was based on TCT measurements as the primary outcome variables. Based on a previous study, the within-subject standard deviation (SD) was 23 [15], and the 95% confidence interval (CI) width was set at ±30% of the within-subject SD. Therefore, the required sample size calculated using the Power Analysis and Sample Size software (i.e., PASS; version 15 NCSS Statistical Software, Kaysville, UT, USA) was 31. Consequently, we aimed to recruit 46 patients to ensure an adequate number of participants. This procedure was established under the guidance of professional medical statisticians.

Thus, 46 patients diagnosed with keratoconus at the Eye and ENT Hospital of Fudan University between January and March 2022 were enrolled in this study. A single eye was randomly selected for enrollment to avoid interference between the eyes. All participants underwent routine ophthalmic examinations, which included tests for uncorrected and best-corrected visual acuity, three-mirror fundus examinations, and slit-lamp microscopy.

### Instruments

The RTVue (RTVue-100, Optovue Inc., Fremont, CA, USA) is a spectral-domain OCT (SD-OCT) device; it is equipped with second-generation OCT technology. It uses a near-infrared, low-coherence, super-luminescent diode light source with a bandwidth of 50 nm and a central wavelength of 830 nm to achieve an axial resolution of 5 μm and a lateral resolution of as high as 1.5 μm in the tissues. Its data acquisition speed is noticeably better than that of the first-generation time-domain OCT (TD-OCT) devices [16].

The Casia-2 (Casia-2, Tomey Corporation, Nagoya, Japan) is the latest swept-source OCT device, and is equipped with a 1310 nm infrared light source with a penetration depth of up to 14 mm in the tissues. It can minimize the influence of measurement light on pupil movement and mydriasis. Therefore, it combines the advantages of single-point detection from TD-OCT with the fast imaging of SD-OCT [14].

The Pentacam (Pentacam HR, Oculus, Wetzlar, Germany) combines a blue light-emitting diode light source with a wavelength of 475 nm with a Scheimpflug camera that rotates 180° to capture 50 slit images in 2 seconds. Each image captures 500 true height points to provide 360° three-dimensional images of the anterior segment [17].

The same trained operator measured all participants using the RTVue, Casia-2, and Pentacam within 4 hours in a dark room, after adjusting for 5 minutes without mydriasis before the examinations. The examinations were not performed in a fixed order; the patients were instructed to hold their heads in place with both eyes open and to look forward. Three consecutive measurements of good quality were performed per eye per device, and one of the three measurements was selected randomly for statistical analyses.

### Statistical analyses

SPSS 26 (IBM Corp., Armonk, NY, USA) and MedCalc version 19.0.4 (MedCalc Software Ltd., Ostend, Belgium) were used for the statistical analyses and plotting. First, a Friedman test and Bonferroni’s multiple comparisons were used to evaluate differences between the CCT, TCT, thinnest point location (X and Y coordinates), and cone deviation measurements among the three devices. The cone deviation was calculated using the Pythagorean theorem for the X and Y Pentacam coordinates of the thinnest point. Then, the data were classified by the C grade for subgroup analyses. Pearson’s correlation coefficients were used to assess CCT and TCT correlations among the three devices. Bland–Altman plots were applied to illustrate CCT and TCT measurement agreements between each method, where the difference between two measurements was plotted against the mean value of the two measurements; 1.96 SD of the difference represented the 95% limits of agreement (LOA). Based on clinical experience, diurnal variations in the corneal thickness, and previous reports, we defined LOAs greater than ±60 μm, between ±30 μm and ± 60 μm, between ±15 μm and ± 30 μm, and less than ±15 μm as indicative of poor, moderate, acceptable, and good agreements, respectively [18].

The factors influencing differences in the OCT and Pentacam measurements were also investigated. The dependent variable was the absolute difference between the OCT and Pentacam corneal thickness measurements. The independent variables were the anterior curvature radius (A) grade; posterior curvature radius (B) grade; corneal pachymetry at thinnest point (C) grade; astigmatism of the anterior corneal surface (measurements above four were obtained from Pentacam); age; cone deviation; difference between the CCT and TCT; and topometric indices from Pentacam including the index of surface variance (ISV), index of vertical asymmetry (IVA), index of height decentration (IHD), index of height asymmetry (IHA), keratoconus index (KI), Radii minimum (Rmin), center keratoconus index (CKI). The difference between the CCT and TCT was based on the mean OCT and Pentacam CCT and TCT measurements. Univariate linear regression was used to screen for potentially related independent variables, and a multivariate linear regression model was built from the relevant variables. The model consisted of no more than four independent variables. A *p*-value of < 0.05 was considered statistically significant.

## Results

### Baseline information

This study included 35 men (76.09%), with 30 right eyes (65.22%); the average age was 24.3 ± 6.2 years. Furthermore, 8 (17.39%), 3 (6.52%), 14 (30.43%), 4 (8.70%), and 17 (36.70%) cases were of stages KC1, KC1–2, KC2, KC2–3, and KC3, respectively.

### Corneal thickness measurement differences

Table 1 presents the corneal thickness measurements for each instrument. The CCT measurement did not differ among the three groups (*p* = 0.434). However, the TCT, thinnest point location, and cone deviation measurements significantly differed among the three groups. The RTVue and Casia-2 TCT measurements were, on average, 15.15 μm and 13.96 μm thinner than the Pentacam TCT measurements, respectively (both *p* < 0.001; Bonferroni’s multiple comparisons). Furthermore, the Y coordinate of the thinnest point significantly differed between the Pentacam and RTVue measurements (*p* = 0.001); the X and Y coordinates of the thinnest point location also significantly differed between the Pentacam and Casia-2 measurements (*p* = 0.015 and *p* < 0.001, respectively), which resulted in a significant difference in the cone deviation measurements between the Pentacam and the two OCT devices (*p* = 0.015 and *p* = 0.005, respectively). The TCT, thinnest point location, and cone deviation measurements obtained by RTVue and Casia-2 did not differ (Table 2).

**Table 1:** Corneal thickness measurements per instrument

Parameter	Mean ± SD	Range
Central corneal thickness (μm)		
RTVue	488.9 ± 41.44	396–560
Casia-2	488.7 ± 42.52	396–560
Pentacam	489.3 ± 44.17	382–570
Thinnest corneal thickness (μm)		
RTVue	465.5 ± 39.78	368–536
Casia-2	466.5 ± 39.39	375–549
Pentacam	480.5 ± 41.27	375–541
Thinnest point (X-coordinate) (mm)		
RTVue	−0.13 ± 0.57	−1.07–1.02
Casia-2	−0.13 ± 0.56	−1.13–0.96
Pentacam	− 0.03 ± 0.45	−0.28–1.34
Thinnest point (Y-coordinate) (mm)		
RTVue	−0.68 ± 0.47	−1.67–0.49
Casia-2	−0.71 ± 0.42	−1.63–0.42
Pentacam	− 0.48 ± 0.30	− 1.45–0.13
Cone deviation (mm)		
RTVue	1.02 ± 0.78	0.00–3.25
Casia-2	0.10 ± 0.69	0.06–3.11
Pentacam	0.96 ± 0.31	0.28–1.81

*Abbreviations*: *SD* Standard deviation

**Table 2:** Corneal thickness measurement differences among the three instruments

		Group 1	Group 2	Group 3
Central corneal thickness	*p*-value	0.434		
Thinnest corneal thickness	Mean diff.	15.15 μm	13.96 μm	−1.91 μm
	*p*-value	< 0.001	< 0.001	0.633
Thinnest point (X-coordinate)	Mean diff.	0.098 μm	0.098 μm	0.000 μm
	*p*-value	0.182	0.015	1.000
Thinnest point (Y-coordinate)	Mean diff.	0.206 mm	0.234 mm	0.028 mm
	*p*-value	0.001	< 0.001	0.891
Cone deviation	Mean diff.	−0.378 mm	−0.359 mm	0.020 mm
	*p*-value	0.015	0.005	1.000

Group 1: Pentacam vs. RTVue; Group 2: Pentacam vs. Casia-2; and Group 3: RTVue vs. Casia-2

Statistical test: Friedman test and Bonferroni multiple comparison

A *p*-value of < 0.05 was considered statistically significant

*Abbreviations*: *Mean diff* mean difference

**Fig. 1:**
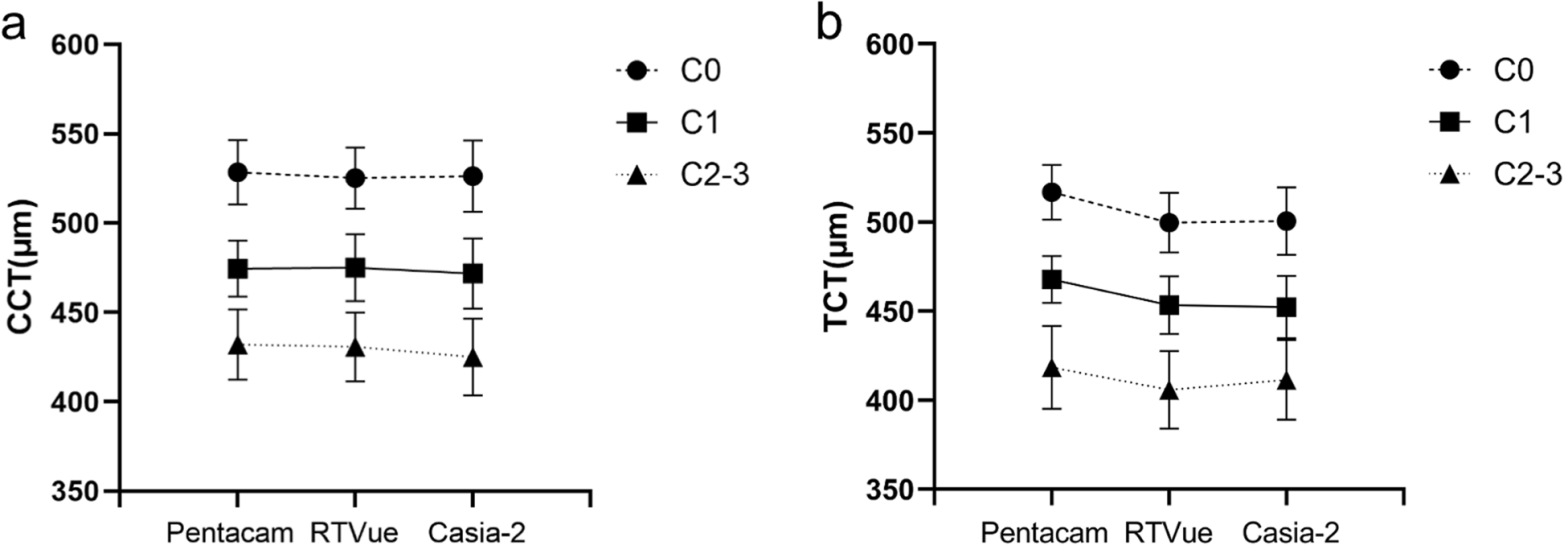
Subgroup analysis of the CCT (**a**) and TCT (**b**) measured by Pentacam, RTVue and Casia-2 based on the C grade. The vertical axis represents the corneal thickness measurements and the horizontal axis shows different devices. Dashed line with round dots represent C0 group. Solid line with square dots represent C1 group. Dot line with triangle dots represent C2–3 group. All scales in um. CCT: Central corneal thickness; TCT: Thinnest corneal thickness

A subgroup analysis was performed based on the C grade (C0, C1, and C2–3). The CCT values of the three subgroups were comparable for all instruments (*p* > 0.05; Fig. 1a). However, the TCT value measured by the Pentacam was consistently higher than that measured by either OCT devices (all *p* < 0.05; Fig. 1b), except for in comparison with the Casia-2 measurements in group C2–3 (*p* = 0.221). Furthermore, as the C grade increased, the difference between the OCT and Pentacam TCT measurements showed a decreasing tendency. Finally, the TCT measurements did not differ between the two OCT devices in any subgroup (Table 3).

**Table 3:** Subgroup analysis of TCT measured by the three instruments according to the C grade

C Grading		Group 1	Group 2	Group 3
C0	Mean diff.	17.05 μm	16.14 μm	− 0.90 μm
	*p*-value	< 0.001	< 0.001	1.000
C1	Mean diff.	14.31 μm	15.44 μm	1.12 μm
	*p*-value	< 0.001	0.001	1.000
C2–3	Mean diff.	12.50 μm	7.00 μm	−5.5 μm
	*p*-value	0.004	0.221	0.438

Group 1: Pentacam vs. RTVue; Group 2: Pentacam vs. Casia-2; and Group 3: RTVue vs. Casia-2

Statistical test: Friedman test and Bonferroni multiple comparison

A *p*-value of < 0.05 was considered statistically significant

*Abbreviations*: *Mean diff* mean difference

### Corneal thickness measurement correlations

For the CCT measurements, the Pearson’s correlation coefficients between Pentacam and RTVue, Pentacam and Casia-2, and RTVue and Casia-2 were 0.981, 0.981, and 0.982, respectively (all *p* < 0.001). For the TCT measurements, the Pearson’s correlation coefficients between Pentacam and RTVue, Pentacam and Casia-2, and RTVue and Casia-2 were 0.975, 0.960, and 0.985, respectively (all *p* < 0.001). These results indicate strong positive correlations among the three devices for the CCT and TCT measurements.

### Corneal thickness measurement agreements

**Fig. 2:**
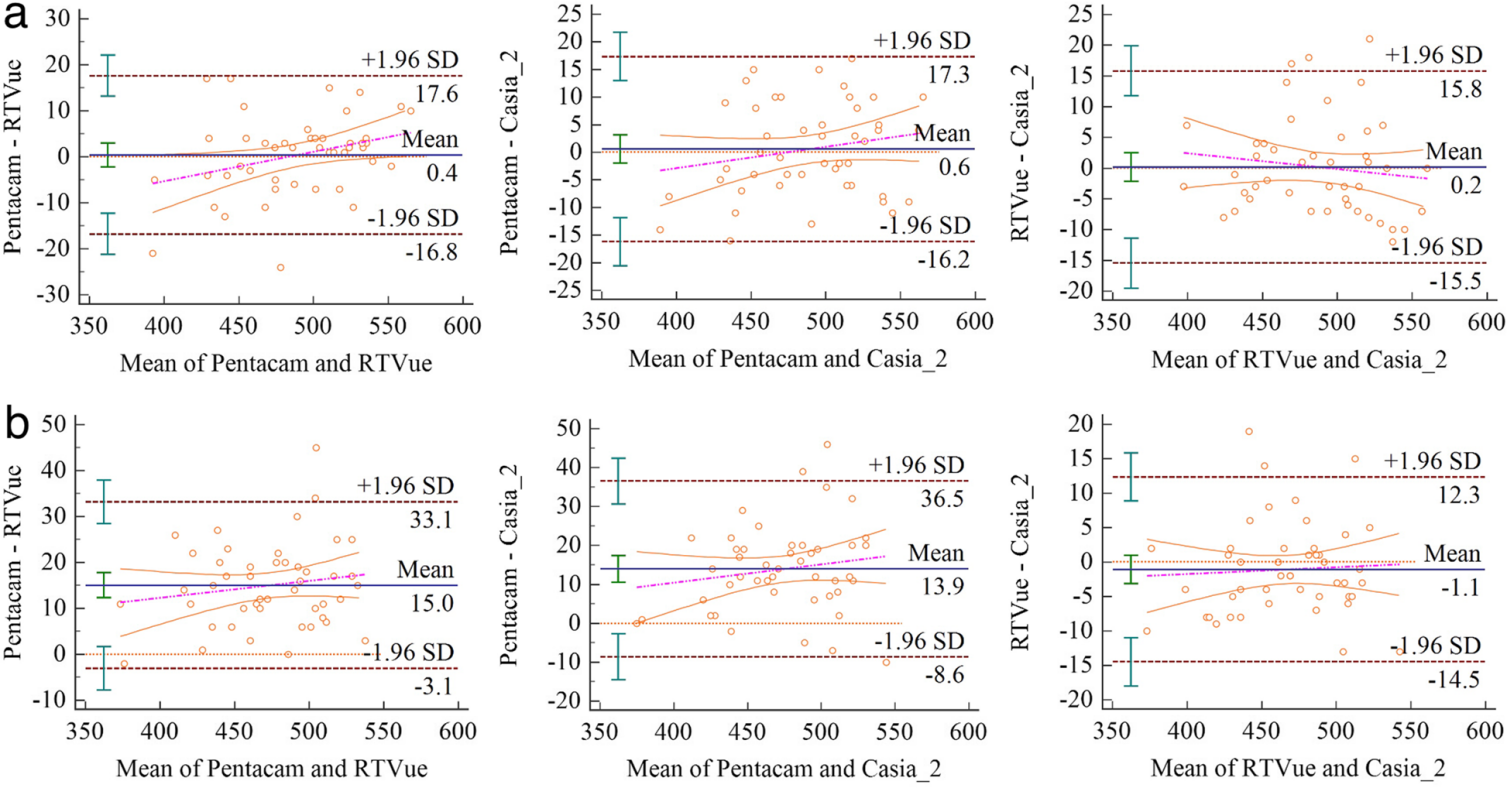
Bland-Altman plots comparing the level of agreement between the 3 instruments for CCT (**a**), TCT (**b**) measurements. The vertical axis represents the difference between these measurements and the horizontal axis shows their mean. Dashed red lines represent the 95% confidence intervals. Solid blue line represents the mean difference. All scales in um. CCT: Central corneal thickness; TCT: Thinnest corneal thickness

For the CCT measurements, the agreements among these devices did not differ (*p* > 0.05; Fig. 2a). For the TCT measurements, the agreement between Pentacam and RTVue and between Pentacam and Casia-2 differed significantly (both *p* < 0.001), with LOAs of − 3.1 μm to + 33.1 μm and − 8.6 μm to + 36.5 μm, respectively. The agreement between RTVue and Casia-2 did not differ (Fig. 2b).

### Factors influencing differences between the Pentacam and OCT measurements

Univariate linear regression analysis revealed that the difference between the Pentacam and RTVue TCT measurements was related to the IHA, CKI, cone deviation, and difference between CCT and TCT. The multivariate linear regression model confirmed that the TCT measurement difference was related to the difference between CCT and TCT (b = 0.490, 95% CI: 0.03 to 0.948, *p* < 0.001). These results indicate that for every 10 μm increase in the difference between CCT and TCT, the TCT measurement difference between the two devices increased by 4.9 μm (Table 4).

**Table 4:** Multivariate linear regression analyses of factors affecting TCT measurement differences among RTVue, Casia-2, and Pentacam

**Pentacam vs. RTVue**	**b**	**95% CI LL**	**95% CI UL**	***p*** **-value**
IHA	−0.61	−0.143	0.022	0.144
CKI	−29.711	−73.020	13.598	0.173
Cone deviation	−4.129	−11.974	3.715	0.294
Difference between CCT and TCT	0.490	0.033	0.948	0.036
**Pentacam vs. Casia-2**	**b**	**95% CI LL**	**95% CI UL**	***p*** **-value**
A grade	3.9	1.753	6.074	0.001
C grade	−7.875	−11.404	−4.346	< 0.001
Difference between CCT and TCT	0.425	0.1	0.751	0.012

Statistical test: Multivariate linear regression

A *p*-value of < 0.05 was considered statistically significant

*Abbreviations*: *CI* confidence interval, *CCT* central corneal thickness, *LL* lower limit, *TCT* thinnest corneal thickness, *UL* upper limit, *IHA* index of height asymmetry, *CKI* center keratoconus index

The univariate and multivariate linear regression analyses indicated that the difference between the Pentacam and Casia-2 TCT measurements was associated with the A grade (b = 3.9, 95% CI: 1.753 to 6.074, *p* = 0.001), C grade (b = − 7.875, 95% CI: − 11.404 to − 4.346, *p* < 0.001), and the difference between CCT and TCT (b = 0.425, 95% CI: 0.1 to 0.751, *p* = 0.012). The TCT measurement difference increased by 3.9 μm for each grade A increase, decreased by 7.875 μm for each grade C increase, and increased by 4.25 μm for each 10 μm increase in the difference between CCT and TCT (Table 4).

## Discussion

Corneal thickness measurements are becoming increasingly important, especially in cases of keratoconus, where the corneal thickness is essential to the diagnosis and treatment. Meanwhile, corneal parameters determined solely from the widely used Pentacam can no longer satisfy the clinical need for detection of early keratoconus changes and imaging through corneal scars; this calls for compensation from FD-OCT devices, such as RTVue and Casia-2. Therefore, exploring three non-contact methods for measuring the corneal thickness in patients with mild-to-moderate keratoconus is of practical importance. This study is the first attempt to compare three measurement devices for corneal thickness with differing working mechanisms to determine if they can be used interchangeably in this patient population.

We found that the CCT measurements obtained by Pentacam, RTVue, and Casia-2 were highly correlated and did not differ among the instruments. We used Bland–Altman plots to illustrate the agreements among the three devices for CCT measurements; an acceptable agreement was noted for all (±15 μm < LOA < ±30 μm). However, most previous studies on patients with keratoconus have reported that compared to Pentacam, FD-OCT tends to underestimate CCT [17, 19]. Patient selection may explain the discrepancy between these previous results and our present findings; previous studies included patients with keratoconus at a wide range of stages, including forme fruste or KC3–4 keratoconus. Furthermore, corneal thickness is obtained by measuring the radial distance between two concentric spheres [12]. However, the anterior and posterior corneal surfaces are neither spherical nor concentric; therefore, if the patient’s head deviates, the devices will measure along a different axis and produce different values. In addition, differences in the device type, age, and sex can affect the CCT measurements [20]. In this study, to ensure high-quality data, we excluded patients with severe keratoconus. Furthermore, to avoid interference between the eyes, we included only a single eye; thus, data on the part of the eye with forme fruste keratoconus were excluded to ensure minimal clinical heterogeneity.

Correlations among the three devices were good for the TCT measurements, but the RTVue TCT measurement was 15.15 μm thinner and the Casia-2 TCT measurement was 13.96 μm thinner than that obtained with the Pentacam. Moreover, the thinnest point location determined by these devices significantly differed from that determined by Pentacam. A previous study reported that compared to Pentacam, Casia-2 underestimated the TCT in keratoconus [14]. This is consistent with our results; there is no similar report for RTVue. To our knowledge, ours is the first study to compare the differences in the measurements of the thinnest point location of the cornea between FD-OCT devices and Pentacam. The difference in the thinnest point location between the devices may explain the difference in the TCT measurements but not in the CCT measurements. For diagnosing and treating keratoconus, a more conservative corneal thickness estimation, such as that provided by OCT, might be necessary for ensuring safety. For example, accurate corneal thickness measurements before and during corneal collagen cross-linking are required to ensure that the cornea is thicker than the safety limit of 400 μm, in order to avoid damage to the corneal endothelium [21]. Accurate corneal thickness measurements also enable optimal decisions on the trephination depth during deep lamellar keratoplasty [22] and replacement corneal stroma implantations [21].

The Bland–Altman plots indicated a relatively wide LOA between RTVue (− 3.1 μm to + 33.1 μm) and Casia-2 (− 8.6 μm to + 36.5 μm) versus Pentacam for TCT measurements with a moderate agreement (±15 μm < LOA < ±30 μm). Therefore, in clinical practice, we recommend using the same device for patients with mild-to-moderate keratoconus, especially for monitoring corneal thickness changes during follow-up. Overall, RTVue and Casia-2 have good agreement for CCT and TCT measurements. Therefore, either device should be selected as a routine supplement to other keratoconus examinations. However, FD-OCT is currently limited in its application to specific ectasia screening tools; such tools and an applicable conversion factor for thickness measurement between Pentcam and FD-OCT should be developed to compensate for the disadvantages of Pentacam, especially in patients with poor ocular surface conditions.

Univariate linear regression analyses revealed that the TCT measurement differences between RTVue and Pentacam were related to the IHA, CKI, cone deviation, and difference between CCT and TCT. However, multivariate linear regression analysis only confirmed the last one. As such, for every 10 μm increase in the difference between CCT and TCT, the TCT deviation between the two devices increases by 4.9 μm. This may be because cone deviation indirectly affects the TCT measurement by affecting the difference between CCT and TCT. Therefore, the TCT measurement is biased, since the thinnest point location differs between the devices, which affects the difference between CCT and TCT.

Univariate and multivariate linear regression analyses also found that the TCT measurement differences between Casia-2 and Pentacam were related to the A grade, C grade, and the difference between CCT and TCT. The difference in the TCT measurement increased by 3.9 μm for each A grade increase and by 4.25 μm for each 10-μm increase in the difference between CCT and TCT. This indicates that these discrepancies are primarily due to distortion of the anterior corneal surface, potentially causing inaccurate positioning of the thinnest point of the cornea and deviations in the measured TCT values. The multifactorial analysis results regarding the C grade corresponded to those of our subgroup analysis of TCT based on the C grade; the results showed that the Pentacam and OCT differences decreased as the C grade increased. Overall, compared with FD-OCT, Pentacam overestimates TCT. However, Pentacam overestimates the corneal thickness for thin corneas to a lesser extent, so the difference with FD-OCT decreases, which may explain our result. However, it does not mean that the agreement between the two increases in thin corneas; studies have also reported that the corneal thickness measured by Pentacam was even lower than that measured by OCT in thin and flat corneas after laser-assisted in situ keratomileusis surgery, which may be related to the decreased reliability of Pentacam in thin corneas [23].

This study has the following limitations. First, the sample size was small and could be expanded for future studies. In addition, only thicknesses of the central corneal region were studied; therefore, the OCT and Pentacam agreements in paracentral corneal thickness measurements should be evaluated in the future. Finally, only patients with primary keratoconus were included. For future studies, comparison between Pentacam and OCTs should be carried out in forme fruste keratoconus and keratoconus suspected cases, in order to improve the diagnostic ability in those cases in avoid of post refractive corneal ectasia as well as to ensure early medical intervention.

## Conclusions

The RTVue, Casia-2, and Pentacam devices had a good agreement for CCT measurement, but not for TCT and the thinnest point location, measurements in patients with mild-to-moderate keratoconus. TCT measurement differences between the OCT devices and the Pentacam are more pronounced in keratoconus cases with a steeper anterior surface, thicker TCT, and a larger difference between the CCT and TCT measurements.

## Supplementary Information

**Additional file 1.**

## Data Availability

The datasets used and/or analyzed during the current study are available from the corresponding author on reasonable request.

## Abbreviations

CCT: Central corneal thickness
TCT: Thinnest corneal thickness
OCT: Optical coherence tomography
FD-OCT: Fourier-domain optical coherence tomography
SD: Standard deviation
CI: Confidence interval
SD-OCT: Spectral-domain optical coherence tomography
TD-OCT: Time-domain optical coherence tomography
LOA: Limits of agreement
IHA: Index of height asymmetry
Rmin: Radii minimum
CKI: Center keratoconus index
